# Supplementary material for: Genetic landscape of prostate cancer conspicuity on multiparametric MRI: a protocol for a systematic review and bioinformatic analysis
Source: BMJ Open. 2020 Jan 27;10(1):e034611. doi: 10.1136/bmjopen-2019-034611 (PMC7045175; doi:10.1136/bmjopen-2019-034611)
Supplement: Author's manuscript [file bmjopen-2019-034611.draft_revisions.pdf]

BMJ Open is committed to open peer review. As part of this commitment we make the peer review history of every article we publish publicly available.

When an article is published we post the peer reviewers' comments and the authors' responses online. We also post the versions of the paper that were used during peer review. These are the versions that the peer review comments apply to.

The versions of the paper that follow are the versions that were submitted during the peer review process. They are not the versions of record or the final published versions. They should not be cited or distributed as the published version of this manuscript.

BMJ Open is an open access journal and the full, final, typeset and author-corrected version of record of the manuscript is available on our site with no access controls, subscription charges or pay-per-view fees (http://bmjopen.bmj.com).

If you have any questions on BMJ Open's open peer review process please email info.bmjopen@bmj.com

BMJ Open

The genetic landscape of prostate cancer conspicuity on multiparametric MRI: a protocol for a systematic review and bioinformatic analysis

Journal:	*BMJ Open*
Manuscript ID	bmjopen-2019-034611
Article Type:	Protocol
Date Submitted by the Author:	27-Sep-2019
Complete List of Authors:	Norris, Joseph; University College London, UCL Division of Surgery & Interventional Science<br>Simpson, Benjamin Scott; University College London, UCL Division of Surgery & Interventional Science<br>Parry, Marina; University College London, UCL Cancer Institute<br>Allen, Clare; University College London Hospitals NHS Foundation Trust, Department of Radiology<br>Ball, Rhys; University College London Hospitals NHS Foundation Trust, Department of Pathology<br>Freeman, Alex; University College London Hospitals NHS Foundation Trust, Department of Pathology<br>Kelly, Daniel; Cardiff University, School of Healthcare Sciences<br>Kirkham, Alex; University College London Hospitals NHS Foundation Trust, Department of Radiology<br>Kasivisvanathan, Veeru; University College London, UCL Division of Surgery & Interventional Science<br>Whitaker, Hayley; University College London, UCL Division of Surgery & Interventional Science<br>Emberton, Mark; University College London, UCL Division of Surgery & Interventional Science
Keywords:	Prostate disease < UROLOGY, Magnetic resonance imaging < RADIOLOGY & IMAGING, Cancer genetics < GENETICS, Urological tumours < ONCOLOGY

SCHOLARONE™
Manuscripts

BMJ

I, the Submitting Author has the right to grant and does grant on behalf of all authors of the Work (as defined in the below author licence), an exclusive licence and/or a non-exclusive licence for contributions from authors who are: i) UK Crown employees; ii) where BMJ has agreed a CC-BY licence shall apply, and/or iii) in accordance with the terms applicable for US Federal Government officers or employees acting as part of their official duties; on a worldwide, perpetual, irrevocable, royalty-free basis to BMJ Publishing Group Ltd ("BMJ") its licensees and where the relevant Journal is co-owned by BMJ to the co-owners of the Journal, to publish the Work in this journal and any other BMJ products and to exploit all rights, as set out in our licence.

The Submitting Author accepts and understands that any supply made under these terms is made by BMJ to the Submitting Author unless you are acting as an employee on behalf of your employer or a postgraduate student of an affiliated institution which is paying any applicable article publishing charge ("APC") for Open Access articles. Where the Submitting Author wishes to make the Work available on an Open Access basis (and intends to pay the relevant APC), the terms of reuse of such Open Access shall be governed by a Creative Commons licence – details of these licences and which Creative Commons licence will apply to this Work are set out in our licence referred to above.

Other than as permitted in any relevant BMJ Author's Self Archiving Policies, I confirm this Work has not been accepted for publication elsewhere, is not being considered for publication elsewhere and does not duplicate material already published. I confirm all authors consent to publication of this Work and authorise the granting of this licence.

For peer review only

The Genetic Landscape of Prostate Cancer Conspicuity on Multiparametric MRI: A Protocol for a Systematic Review and Bioinformatic Analysis

Joseph M. Norris,[1,2,3]* Benjamin S. Simpson,[1]* Marina A. Parry,[4] Clare Allen,[6] Rhys Ball,[7] Alex Freeman,[7] Daniel Kelly,[8] Alex Kirkham,[6] Veeru Kasivisvanathan,[1,2,3] Hayley C. Whitaker,[1] Mark Emberton[1,2]

*joint first-name authors

[1] UCL Division of Surgery & Interventional Science, University College London, London, UK

[2] London Deanery of Urology, London, UK

[3] Department of Urology, University College London Hospitals NHS Foundation Trust, London, UK

[4] UCL Cancer Institute, University College London, London, UK

[6] Department of Radiology, University College London Hospitals NHS Foundation Trust, London, UK

[7] Department of Pathology, University College London Hospitals NHS Foundation Trust, London, UK

[8] School of Healthcare Sciences, Cardiff University, Wales, UK

Correspondence to: Mr Joseph M Norris BSc (Hons), MSc, BM BS, MRCS (Eng)

MRC Clinical Research Training Fellow, University College London

UCL Division of Surgery & Interventional Science, London, UK, W1W 7TS

Email: joseph.norris@ucl.ac.uk

Phone: +44 7724022171

ORCID: 0000-0003-2294-0303

Article type: Systematic review protocol.

Word count: 2,448 words

Ethics: Not required.

Conflicts of interest: None to declare.

Funding: Joseph M. Norris is funded by the Medical Research Council (MRC) on an MRC Clinical Research Training Fellowship (MRC Grant Ref: MR/S00680X/1). Benjamin S. Simpson is funded by the Rosetrees Foundation.

Keywords: Bioinformatic analysis, genetics, genomics, multiparametric MRI, prostate cancer, systematic review.

Abbreviations: DWI, diffusion weighted imaging; Gene Expression Omnibus, GEO; mpMRI, multiparametric magnetic resonance imaging; MRC, Medical Research Council; National Center for Biotechnology Information, NCBI; NICE, National Institute for Health and Care Excellence; UK, United Kingdom.

For peer review only - http://bmjopen.bmj.com/site/about/guidelines.xhtml

Introduction

The introduction of multiparametric resonance imaging (mpMRI) has enabled enhanced risk stratification for men at risk of prostate cancer, through accurate pre-biopsy identification of clinically significant disease. However, approximately 10-20% of significant prostate cancer may be missed on mpMRI. It appears that the genomic basis of lesion visibility or invisibility on mpMRI may have key implications for prognosis and treatment. Here, we describe a protocol for the first systematic review and novel bioinformatic analysis of the genomic basis of prostate cancer conspicuity on mpMRI.

Methods and analysis

A systematic search of MEDLINE, PubMed, EMBASE, and Cochrane databases will be conducted. Screening, data extraction, statistical analysis and reporting will be performed in accordance with the Preferred Reporting Items for Systematic Reviews and Meta-Analyses (PRISMA) guidelines. Included papers will be full text articles, comparing molecular characteristics of mpMRI-visible lesions and mpMRI-invisible lesions at the DNA, DNA-methylation, RNA, or protein level. Study bias and quality will be assessed using a modified Newcastle-Ottawa score. Additionally, we will conduct a novel bioinformatic analysis of supplementary material and publicly available data, to combine transcriptomic data and reveal common pathways highlighted across studies. To ensure methodological rigour, this protocol is written in accordance with the PRISMA Protocol 2015 checklist.

Ethics and dissemination

Ethical approval will not be required, as this is an academic review of published literature. Findings will be disseminated through publications in peer-reviewed journals, and presentations at national and international conferences.

PROSPERO registration number CRD42019147423

Strengths and limitations of this study:

- This project will be the first comprehensive review of the molecular characteristics of mpMRI-visible and mpMRI-invisible lesions, following key the methodological steps of the PRISMA guidelines.
- Novel bioinformatic, over-representation analysis will reveal shared pathways and genes involved in prostate cancer mpMRI-conspicuity, that are overlooked by individual studies.
- The degree of heterogeneity between the included studies limits the comparability of derived results.
- There are relatively few studies that directly compare the genomic basis of mpMRI-visible and mpMRI-invisible lesions, which potentially limits the strength of conclusions from this work.

For peer review only - http://bmjopen.bmj.com/site/about/guidelines.xhtml

BACKGROUND

Pre-biopsy multiparametric magnetic resonance imaging (mpMRI) has excellent diagnostic accuracy, and has improved risk stratification in prostate cancer diagnosis, compared to systematic transrectal ultrasound–guided prostate biopsy.[1,2] Despite this, approximately 10-20% of clinically significant prostate cancers remain mpMRI invisible.[1,3,4]

Suspicious mpMRI phenotypes appear to closely associate with the presence of prostate cancer, and to an extent, its Gleason grade.[1] However, the degree to which the molecular landscape of prostate cancer correlates with mpMRI is unclear. In contrast, the general molecular basis of prostate cancer is well studied, with large-scale genomic enquires defining key drivers in tumour development and progression. Aberrations, including, recurrent mutations in SPOP1, FOXA1, IDH1, fusions in TMPRSS/ERG, ETV1/4, FLI1, and copy number alterations, such as MYC amplification or NKX3-1, RB1 and PTEN deletions or transcriptomic changes in AMACR, PCA3, GDF15 and MSMB, have all been investigated in the context of prostate cancer.[5-7] Over the past five years, there has been an increased effort to use this to develop our understanding of the genetic basis of prostate cancer conspicuity on mpMRI. It is now prudent to collate this growing evidence base, to clarify on progress made so far, and identify the most pertinent areas for future research.

The aim of this systematic review and bioinformatic analysis is to determine and summarise for the first time, the genomic correlates of tumour visibility and invisibility on mpMRI, in order to elucidate the mechanisms that underpin mpMRI conspicuity, and the prognostic implication of mpMRI phenotypes.

For peer review only - http://bmjopen.bmj.com/site/about/guidelines.xhtml

METHODS AND ANALYSIS

This protocol of the planned systematic review and bioinformatic analysis is written in-line with the Preferred Reporting Items for Systematic Reviews and Meta-Analyses Protocol (PRISMA-P) 2015 checklist and is registered in the PROSPERO International Registry (PROSPERO Reg. No.: CRD42019147423).[8] Selected studies will be used for thematic synthesis and appropriate supplementary material extracted. In addition to the literature search, a bioinformatic approach will be used to identify relevant publicly available genomic data for a comprehensive analysis of over-represented pathways and biological functions.

Search methodology

Searches will be carried out using the MEDLINE, PubMed, EMBASE, and Cochrane databases in order to retrieve maximum yield of relevant evidence. The search will include Medical Subject Headings (MESH) terms and free text, combined with Boolean operators. The search will include the terms: 'prostate', 'cancer' and 'MRI', as well as multiple synonyms for the term 'genetics', to account for large heterogeneity in nomenclature and diversity of topics encompassed within this heading. Identified articles will be uploaded to Rayyan, a semi-automated tool to expedite the initial screening process and to allow two reviewers to filter duplicate studies and screen articles for relevance.[9] Furthermore, the reference section of all included articles will be searched manually to identify missed studies or additional data. Finally, experts will be consulted to identify additional literature. For the bioinformatic analysis, the National Center for Biotechnology Information (NCBI) Gene Expression Omnibus (GEO) and European Genome-phenome archive will be searched using the same search parameters as those used for the literature search. If sufficient processed data is available, data will be extracted from supplementary to ensure data veracity. In the case of missing data, the corresponding authors will be contacted directly.

Study selection and data extraction

Two researchers will independently screen eligible studies, assessing titles and abstracts for relevance. If considered eligible, the full text will be retrieved and further reviewed for eligibility. Any lack of concordance between reviewers will be discussed until a consensus is reached or passed to a third reviewer. Additionally, the reason for exclusion will be noted for later analysis. This process will be documented in detail in order to generate the PRISMA flow diagram.

Inclusion criteria

To be included in the analysis, studies must investigate one or more genomic aspects of the appearance of prostate cancer on mpMRI. Genomic investigation could be at the DNA level, investigating SNPs, or somatic alterations, or focused on larger scale alterations, such as copy number changes. Moreover, investigations revealing higher order structures, such as methylation, will also be included. Transcriptomic data analysing RNA expression (coding or non-coding) or microRNA will also be included, as will investigation into protein expression.

Exclusion criteria

For peer review only - http://bmjopen.bmj.com/site/about/guidelines.xhtml

Conference abstracts, correspondence articles, expert opinions, and case reports will be excluded. Studies that do not correlate mpMRI phenotypes with genomic data will be excluded. Articles focusing solely on macro-characteristics or clinical features of mpMRI conspicuity will be removed.

Data extraction

All extracted data will be collated in a dedicated data sheet. Both reviewers will extract data independently from each other and agree on consensus. Table 1 summarises data items to be collected.

Table 1 Data collection items

Item no.	Data title	Data type
1	Year of publication	Study characteristic
2	Study authors	Study characteristic
3	Study design	Study characteristic
4	Patient population	Demographics
5	Number of participants	Demographics
6	mpMRI scoring scheme used	Methodology
7	Definition for clinically significant disease	Methodology
8	Definition for lesion visibility & invisibility	Methodology
9	Sample processing approach	Methodology
10	Biomolecule studied	Outcome
11	Differential expression of biomolecule	Outcome

mpMRI, multiparametric magnetic resonance imaging; no., number.

Endpoints

The primary endpoint will be differential gene expression between mpMRI-visible and mpMRI-invisible lesions. Secondary endpoints will include explanatory links between gene function and mpMRI-conspicuity, and the prognostic value of differential gene enrichment.

Sub-group analysis

A secondary analysis will look at whether gene panels, or individual genes, associated with mpMRI-conspicuity show different expression levels when key attributes (for example, Gleason grade and tumour size) are matched between study arms.

For peer review only - http://bmjopen.bmj.com/site/about/guidelines.xhtml

Statistical analysis

For the bioinformatic analysis, we will aim to keep data as close to the original format that was provided in the selected study. In the case of transcriptomic data, we will extract Log2 fold change and the associated false discovery rate (FDR) adjusted value between mpMRI-visible and mpMRI-invisible tumours. In more complex comparisons, we will attempt to extract details which indicate concordance of differential gene expression between studies, however, we will not include effect size if this is incomparable. If unavailable, we will simply compare highlighted genomic features and the direction of change between groups. Genes highlighted in multiple studies will be used (via over-representation analysis) to identify enriched pathways. This method does not rely on effect size weighting, and uses a modified Fisher's exact test to identify enriched biological processes.[10] Steps in the bioinformatic analysis are outlined in table 2.

Table 2 Steps in the bioinformatic analysis

Step no.	Task
**Step 1**	Identifying studies with suitable supplementary data or associated data in data repositories
**Step 2**	Assessing comparability of results
**Step 3**	Comparing overlapping genes in multiple studies
**Step 4**	Over-representation analysis of genes present in multiple studies
**Step 5**	Comparison of suggested gene panels in independent cohort datasets

No., number.

Risk of bias in individual studies

To assess bias and quality across the included studies, a modified Newcastle-Ottawa score will be used, designed to assess observational cohort studies.[11] This scoring system is split into three main sections: selection, comparability, and outcome. Each section contains sub-questions that assess the quality of the research methodology, at the study level. Two reviewers will be involved with this process, and any disagreement will be settled by consensus. The results of the bias assessment will be used to influence data synthesis by providing an assessment of the reliability and applicability of the data produced. If studies are found to be of low quality (or high bias) then they may be excluded, or if included, they will be accompanied by appropriate commentary in the discussion. As this review centres around genomic studies of conspicuity as opposed to treatment outcome, non-applicable sections may be modified to reflect the evidence base, and reduce reporting inaccuracy.

For peer review only - http://bmjopen.bmj.com/site/about/guidelines.xhtml

DISCUSSION

In the UK, the National Institute for Health and Care Excellence (NICE) have recently updated their guidelines for men with suspected prostate cancer.[12] Men with suspected prostate cancer should now undergo mpMRI before prostate biopsy, as part of their risk stratification.[13] This move will likely see a rise in the numbers of mpMRI performed, and as such, we should strive to better understand the nature of disease detected and missed by this technology. Through systematic review and bioinformatic analysis, we aim to identify commonality between genomic-based studies which have investigated mpMRI-visible and mpMRI-invisible tumours. Our results will enhance the understanding of the genomic basis of prostate cancer visibility on mpMRI, the pathways that underpin this, and ultimately, the prognostic implication of disease conspicuity.

In our thematic analyses, we aim to derive the most prominent themes from studies that compare genetic differences in mpMRI-visible and mpMRI-invisible disease. The mechanistic links between genomic characteristics and the appearance of prostate cancer on mpMRI are not clear, however, early evidence suggests that genes controlling tumour cell proliferation may impact on perfusion, and as such, generation of mpMRI signal, particularly on diffusion weighted imaging (DWI) sequences.[14] Furthermore, combining suitable studies that link tumour conspicuity to genetic control will strengthen our understanding. Another important potential theme that we aim to elicit is the prognostic implication of different mpMRI-visibility or mpMRI-invisibility phenotypes (as defined by PI-RADS or Likert scoring schemes).[15] It appears that mpMRI-visible prostate cancer tends to have higher expression of genes associated with poor prognosis, however, this view is not unanimous throughout the literature and our rigorous systematic analysis will expose key differences in expression of prognostically significant genes, across different studies.[16,17]

In our bioinformatic analyses, we will compare large, publicly available genomic datasets that are linked to included studies from our systematic review. Described by Weidman and Arrison as 'data exploitation,' this approach allows effective use of collective information to obtain new insights.[18] We hope to elucidate commonalities between the strongest molecular differences between mpMRI-visible and mpMRI-invisible tumours. Pooling of data will strengthen association of particular genes with previously described features (for example, molecular pathways and prognostic value). Furthermore, bioinformatic comparison of these datasets may reveal potential key targets for future research, including the development of peripheral biomarkers to identify clinically significant, mpMRI-inconspicuous prostate cancer.

In summary, this systematic review and novel bioinformatic analysis of publicly available genomic data will collate extant evidence in this emerging field, for the first time. Synthesis of these studies will enhance our current understanding of the role that genetics play in the mpMRI-conspicuity of prostate cancer, and will clarify the most pertinent areas for future research.

For peer review only - http://bmjopen.bmj.com/site/about/guidelines.xhtml

Trial status

► Preliminary searches: started.

► Piloting of the study selection process: started.

► Formal screening: started.

► Data extraction: not started.

► Risk of bias assessment: not started.

► Data analysis: not started.

Draft of search strategy for MEDLINE, EMBASE, PubMed and Cochrane Databases

(prostate AND cancer) AND (gene OR genetic OR genome OR genomic OR transcriptome OR transcriptomic OR epigenetic) AND (magnetic resonance imaging OR MRI)

Acknowledgements

None.

Patient and public involvement

Patients and the public were not involved in the development of this systematic review protocol.

For peer review only

For peer review only - http://bmjopen.bmj.com/site/about/guidelines.xhtml

ETHICS AND DISSEMINATION

Due to the nature of the study, there are no relevant ethical concerns and informed consent will not be required. The protocol and systematic review will be disseminated via a peer-reviewed journal.

Author affiliations

[1] UCL Division of Surgery and Interventional Science, University College London, London, UK

[2] London Deanery of Urology, London, UK

[3] Department of Urology, University College London Hospitals NHS Foundation Trust, London, UK

[4] UCL Cancer Institute, University College London, London, UK

[5] Department of Urology, Frimley Health NHS Foundation Trust, London, UK

[6] Department of Radiology, University College London Hospitals NHS Foundation Trust, London, UK

[7] Department of Pathology, University College London Hospitals NHS Foundation Trust, London, UK

[8] School of Healthcare Sciences, Cardiff University, Wales, UK

Contributors

The authors' contribution includes, but is not limited to, the following: JMN and BSS drafted the manuscript and created the study concept. MAP gave advice on genetic aspects. AK, AF, DK, VK, HW and ME provided supervision and guidance during the study. All authors reviewed and approved the manuscript in its current form.

Funding

Joseph M. Norris is funded by the Medical Research Council (MRC) on an MRC Clinical Research Training Fellowship (MRC Grant Ref: MR/S00680X/1). Benjamin S. Simpson receives funding from the Rosetrees Foundation.

Competing interests

None declared.

Patient consent for publication

Not required.

Provenance and peer review

Not commissioned; externally peer-reviewed.

Open access

This is an Open Access article distributed in accordance with the Creative Commons Attribution Non Commercial (CC BY-NC 4.0) license, which permits others to distribute, remix, adapt, build upon this work non-commercially, and license their derivative works on different terms, provided the original work is properly cited and the use is non-commercial. See: http://creativecommons.org/ licenses/by-nc/4.0/

For peer review only - http://bmjopen.bmj.com/site/about/guidelines.xhtml

© Article author(s) (or their employer(s) unless otherwise stated in the text of the article) 2019. All rights reserved. No commercial use is permitted unless otherwise expressly granted.

For peer review only

REFERENCES

1. Ahmed HU, El-Shater Bosaily A, Brown LC, Gabe R, Kaplan R, Parmar MK, Collaco-Moraes Y, Ward K, Hindley RG, Freeman A, Kirkham AP, Oldroyd R, Parker C, Emberton M; PROMIS study group. Diagnostic accuracy of multi-parametric MRI and TRUS biopsy in prostate cancer (PROMIS): a paired validating confirmatory study. Lancet. 2017 Feb 25;389(10071):815-22.
2. Kasivisvanathan V, Rannikko AS, Borghi M, Panebianco V, Mynderse LA, Vaarala MH, Briganti A, Budäus L, Hellawell G, Hindley RG, Roobol MJ, Eggener S, Ghei M, Villers A, Bladou F, Villeirs GM, Virdi J, Boxler S, Robert G, Singh PB, Venderink W, Hadaschik BA, Ruffion A, Hu JC, Margolis D, Crouzet S, Klotz L, Taneja SS, Pinto P, Gill I, Allen C, Giganti F, Freeman A, Morris S, Punwani S, Williams NR, Brew-Graves C, Deeks J, Takwoingi Y, Emberton M, Moore CM; PRECISION Study Group Collaborators. MRI-targeted or standard biopsy for prostate-cancer diagnosis. N Engl J Med. 2018 May 10;378(19):1767-77.
3. Radtke JP, Kuru TH, Boxler S, Alt CD, Popeneciu IV, Huettenbrink C, Klein T, Steinemann S, Bergstraesser C, Roethke M, Roth W, Schlemmer HP, Hohenfellner M, Hadaschik BA. Comparative analysis of transperineal template saturation prostate biopsy versus magnetic resonance imaging targeted biopsy with magnetic resonance imaging-ultrasound fusion guidance. J Urol. 2015 Jan;193(1):87-94.
4. Drost FH, Osses DF, Nieboer D, Steyerberg EW, Bangma CH, Roobol MJ, Schoots IG. Prostate MRI, with or without MRI-targeted biopsy, and systematic biopsy for detecting prostate cancer. Cochrane Database Syst Rev. 2019 Apr 25;4:CD012663.
5. Taylor BS, Schultz N, Hieronymus H, Gopalan A, Xiao Y, Carver BS, Arora VK, Kaushik P, Cerami E, Reva B, Antipin Y. Integrative genomic profiling of human prostate cancer. Cancer Cell. 2010 Jul 13;18(1):11-22.
6. Ross-Adams H, Lamb AD, Dunning MJ, Halim S, Lindberg J, Massie CM, Egevad LA, Russell R, Ramos-Montoya A, Vowler SL, Sharma NL, Kay J, Whitaker H, Clark J, Hurst R, Gnanapragasam VJ, Shah NC, Warren AY, Cooper CS, Lynch AG, Stark R, Mills IG, Grönberg H, Neal DE; CamCaP Study Group. Integration of copy number and transcriptomics provides risk stratification in prostate cancer: a discovery and validation cohort study. EBioMedicine. 2015 Jul 29;2(9):1133-44.
7. Abeshouse A, Ahn J, Akbani R, Ally A, Amin S, Andry CD, Annala M, Aprikian A, Armenia J, Arora A, Auman JT. The molecular taxonomy of primary prostate cancer. Cell. 2015 Nov 5;163(4):1011-25.
8. Moher D, Shamseer L, Clarke M, Ghersi D, Liberati A, Petticrew M, Shekelle P, Stewart LA; PRISMA-P Group. Preferred reporting items for systematic review and meta-analysis protocols (PRISMA-P) 2015 statement. Syst Rev. 2015 Jan 1;4:1.
9. Ouzzani M, Hammady H, Fedorowicz Z, Elmagarmid A. Rayyan—a web and mobile app for systematic reviews. 2016 Dec 5;5(1):210.
10. Khatri P, Sirota M, Butte AJ. Ten years of pathway analysis: current approaches and outstanding challenges. PLoS Comput Biol. 2012;8:e1002375.
11. Wells G, Shea B, Oconnell D . The Newcastle-Ottawa scale (NOS) for assessing the quality of nonrandomized studies in meta-analyses. Available from: http://www.ohri.ca/programs/clinical_epidemiology/oxford.asp
12. Wise J. NICE recommends MRI for suspected prostate cancer to reduce biopsies. BMJ. 2018; 363:k5290.
13. Dasgupta P, Davis J, Hughes S. NICE guidelines on prostate cancer 2019. BJU Int. 2019 Jul;124(1):1.
14. Li P, You S, Nguyen C, Wang Y, Kim J, Sirohi D, Ziembiec A, Luthringer D, Lin SC, Daskivich T, Wu J, Freeman MR, Saouaf R, Li D, Kim HL. Genes involved in prostate cancer progression determine MRI visibility. Theranostics. 2018 Feb 12;8(7):1752-65.

For peer review only - http://bmjopen.bmj.com/site/about/guidelines.xhtml

15. Padhani AR, Barentsz J, Villeirs G, Rosenkrantz AB, Margolis DJ, Turkbey B, Thoeny HC, Cornud F, Haider MA, Macura KJ, Tempany CM, Verma S, Weinreb JC. PI-RADS steering committee: the PI-RADS multiparametric MRI and MRI-directed biopsy pathway. Radiology. 2019 Aug;292(2):464-7

16. Houlahan KE, Salmasi A, Sadun TY, Pooli A, Felker ER, Livingstone J, Huang V, Raman SS, Ahuja P, Sisk AE Jr, Boutros PC, Reiter RE. Molecular hallmarks of multiparametric magnetic resonance imaging visibility in prostate cancer. Eur Urol. 2019 Jul;76(1):18-23.

17. Parry MA, Srivastava S, Ali A, Cannistraci A, Antonello J, Barros-Silva JD, Ubertini V, Ramani V, Lau M, Shanks J, Nonaka D, Oliveira P, Hambrock T, Leong HS, Dhomen N, Miller C, Brady G, Dive C, Clarke NW, Marais R, Baena E. Genomic evaluation of multiparametric magnetic resonance imaging-visible and -nonvisible lesions in clinically localised prostate cancer. Eur Urol Oncol. 2019 Feb;2(1):1-11.

18. Weidman S, Arrison T. Steps toward large-scale data integration in the sciences: summary of a workshop. National Research Council of the National Academies. 2010.

For peer review only

For peer review only - http://bmjopen.bmj.com/site/about/guidelines.xhtml

PRISMA-P (Preferred Reporting Items for Systematic review and Meta-Analysis Protocols) 2015 checklist: recommended items to address in a systematic review protocol*

	Section and topic	Item No	Checklist item
	**ADMINISTRATIVE INFORMATION**		
	Title:		
	Identification	1a	Identify the report as a protocol of a systematic review
✓	Update	1b	If the protocol is for an update of a previous systematic review, identify as such
	Registration	2	If registered, provide the name of the registry (such as PROSPERO) and registration number
	Authors:		
✓	Contact	3a	Provide name, institutional affiliation, e-mail address of all protocol authors; provide physical mailing address of corresponding author
	Contributions	3b	Describe contributions of protocol authors and identify the guarantor of the review
✓	Amendments	4	If the protocol represents an amendment of a previously completed or published protocol, identify as such and list changes; otherwise, state plan for documenting important protocol amendments
	Support:		
	Sources	5a	Indicate sources of financial or other support for the review
✓	Sponsor	5b	Provide name for the review funder and/or sponsor
	Role of sponsor or funder	5c	Describe roles of funder(s), sponsor(s), and/or institution(s), if any, in developing the protocol
	**INTRODUCTION**		
	Rationale	6	Describe the rationale for the review in the context of what is already known
✓	Objectives	7	Provide an explicit statement of the question(s) the review will address with reference to participants, interventions, comparators, and outcomes (PICO)
	**METHODS**		
✓	Eligibility criteria	8	Specify the study characteristics (such as PICO, study design, setting, time frame) and report characteristics (such as years considered, language, publication status) to be used as criteria for eligibility for the review
✓	Information sources	9	Describe all intended information sources (such as electronic databases, contact with study authors, trial registers or other grey literature sources) with planned dates of coverage
✓	Search strategy	10	Present draft of search strategy to be used for at least one electronic database, including planned limits, such that it could be repeated
	Study records:		
✓	Data management	11a	Describe the mechanism(s) that will be used to manage records and data throughout the review

For peer review only

	Selection process	11b	State the process that will be used for selecting studies (such as two independent reviewers) through each phase of the review (that is, screening, eligibility and inclusion in meta-analysis)
✓	Data collection process	11c	Describe planned method of extracting data from reports (such as piloting forms, done independently, in duplicate), any processes for obtaining and confirming data from investigators
	Data items	12	List and define all variables for which data will be sought (such as PICO items, funding sources), any pre-planned data assumptions and simplifications
✓	Outcomes and prioritization	13	List and define all outcomes for which data will be sought, including prioritization of main and additional outcomes, with rationale
✓	Risk of bias in individual studies	14	Describe anticipated methods for assessing risk of bias of individual studies, including whether this will be done at the outcome or study level, or both; state how this information will be used in data synthesis
	Data synthesis	15a	Describe criteria under which study data will be quantitatively synthesised
✓		15b	If data are appropriate for quantitative synthesis, describe planned summary measures, methods of handling data and methods of combining data from studies, including any planned exploration of consistency (such as I^2, Kendall's τ)
		15c	Describe any proposed additional analyses (such as sensitivity or subgroup analyses, meta-regression)
		15d	If quantitative synthesis is not appropriate, describe the type of summary planned
✓	Meta-bias(es)	16	Specify any planned assessment of meta-bias(es) (such as publication bias across studies, selective reporting within studies)
	Confidence in cumulative evidence	17	Describe how the strength of the body of evidence will be assessed (such as GRADE)

*** It is strongly recommended that this checklist be read in conjunction with the PRISMA-P Explanation and Elaboration (cite when available) for important clarification on the items. Amendments to a review protocol should be tracked and dated. The copyright for PRISMA-P (including checklist) is held by the PRISMA-P Group and is distributed under a Creative Commons Attribution Licence 4.0.**

From: Shamseer L, Moher D, Clarke M, Ghersi D, Liberati A, Petticrew M, Shekelle P, Stewart L, PRISMA-P Group. Preferred reporting items for systematic review and meta-analysis protocols (PRISMA-P) 2015: elaboration and explanation. BMJ. 2015 Jan 2;349(jan02 1):g7647.

For peer review only

The genetic landscape of prostate cancer conspicuity on multiparametric MRI: a protocol for a systematic review and bioinformatic analysis

Journal:	*BMJ Open*
Manuscript ID	bmjopen-2019-034611.R1
Article Type:	Protocol
Date Submitted by the Author:	30-Dec-2019
Complete List of Authors:	Norris, Joseph; University College London, UCL Division of Surgery & Interventional Science<br>Simpson, Benjamin Scott; University College London, UCL Division of Surgery & Interventional Science<br>Parry, Marina; University College London, UCL Cancer Institute<br>Allen, Clare; University College London Hospitals NHS Foundation Trust, Department of Radiology<br>Ball, Rhys; University College London Hospitals NHS Foundation Trust, Department of Pathology<br>Freeman, Alex; University College London Hospitals NHS Foundation Trust, Department of Pathology<br>Kelly, Daniel; Cardiff University, School of Healthcare Sciences<br>Kirkham, Alex; University College London Hospitals NHS Foundation Trust, Department of Radiology<br>Kasivisvanathan, Veeru; University College London, UCL Division of Surgery & Interventional Science<br>Whitaker, Hayley; University College London, UCL Division of Surgery & Interventional Science<br>Emberton, Mark; University College London, UCL Division of Surgery & Interventional Science
**Primary Subject Heading**:	Urology
Secondary Subject Heading:	Oncology, Radiology and imaging, Genetics and genomics, Evidence based practice
Keywords:	Prostate disease < UROLOGY, Magnetic resonance imaging < RADIOLOGY & IMAGING, Cancer genetics < GENETICS, Urological tumours < ONCOLOGY

I, the Submitting Author has the right to grant and does grant on behalf of all authors of the Work (as defined in the below author licence), an exclusive licence and/or a non-exclusive licence for contributions from authors who are: i) UK Crown employees; ii) where BMJ has agreed a CC-BY licence shall apply, and/or iii) in accordance with the terms applicable for US Federal Government officers or employees acting as part of their official duties; on a worldwide, perpetual, irrevocable, royalty-free basis to BMJ Publishing Group Ltd ("BMJ") its licensees and where the relevant Journal is co-owned by BMJ to the co-owners of the Journal, to publish the Work in this journal and any other BMJ products and to exploit all rights, as set out in our licence.

The Submitting Author accepts and understands that any supply made under these terms is made by BMJ to the Submitting Author unless you are acting as an employee on behalf of your employer or a postgraduate student of an affiliated institution which is paying any applicable article publishing charge ("APC") for Open Access articles. Where the Submitting Author wishes to make the Work available on an Open Access basis (and intends to pay the relevant APC), the terms of reuse of such Open Access shall be governed by a Creative Commons licence – details of these licences and which Creative Commons licence will apply to this Work are set out in our licence referred to above.

Other than as permitted in any relevant BMJ Author's Self Archiving Policies, I confirm this Work has not been accepted for publication elsewhere, is not being considered for publication elsewhere and does not duplicate material already published. I confirm all authors consent to publication of this Work and authorise the granting of this licence.

For peer review only

The Genetic Landscape of Prostate Cancer Conspicuity on Multiparametric MRI:

A Protocol for a Systematic Review and Bioinformatic Analysis

Joseph M. Norris,[1,2,3] Benjamin S. Simpson,[1]* Marina A. Parry,[4] Clare Allen,[6] Rhys Ball,[7] Alex Freeman,[7]*

Daniel Kelly,[8] Alex Kirkham,[6] Veeru Kasivisvanathan,[1,2,3] Hayley C. Whitaker,[1] Mark Emberton[1,2]

**joint first-name authors*

[1] UCL Division of Surgery and Interventional Science, University College London, London, UK

[2] London Deanery of Urology, London, UK

[3] Department of Urology, University College London Hospitals NHS Foundation Trust, London, UK

[4] UCL Cancer Institute, University College London, London, UK

[6] Department of Radiology, University College London Hospitals NHS Foundation Trust, London, UK

[7] Department of Pathology, University College London Hospitals NHS Foundation Trust, London, UK

[8] School of Healthcare Sciences, Cardiff University, Wales, UK

Correspondence to: *Mr Joseph M Norris BSc (Hons), MSc, BM BS, MRCS (Eng)*

MRC Clinical Research Training Fellow, University College London

UCL Division of Surgery & Interventional Science, London, UK, W1W 7TS

Email: joseph.norris@ucl.ac.uk

Phone: +44 7724022171

ORCID: 0000-0003-2294-0303

Article type: Systematic review protocol.

Word count: 2,284 words

Ethics: Not required.

Conflicts of interest: None to declare.

Funding: Joseph M. Norris is funded by the Medical Research Council (MRC) on an MRC Clinical Research Training Fellowship (MRC Grant Ref: MR/S00680X/1). Benjamin S. Simpson is funded by the Rosetrees Foundation.

Keywords: Bioinformatic analysis, genetics, genomics, multiparametric MRI, prostate cancer, systematic review.

Abbreviations: DWI, diffusion weighted imaging; Gene Expression Omnibus, GEO; mpMRI, multiparametric magnetic resonance imaging; MRC, Medical Research Council; National Center for Biotechnology Information, NCBI; NICE, National Institute for Health and Care Excellence; UK, United Kingdom.

For peer review only

For peer review only - http://bmjopen.bmj.com/site/about/guidelines.xhtml

Introduction

The introduction of multiparametric resonance imaging (mpMRI) has enabled enhanced risk stratification for men at risk of prostate cancer, through accurate pre-biopsy identification of clinically significant disease. However, approximately 10-20% of significant prostate cancer may be missed on mpMRI. It appears that the genomic basis of lesion visibility or invisibility on mpMRI may have key implications for prognosis and treatment. Here, we describe a protocol for the first systematic review and novel bioinformatic analysis of the genomic basis of prostate cancer conspicuity on mpMRI.

Methods and analysis

A systematic search of MEDLINE, PubMed, EMBASE, and Cochrane databases will be conducted. Screening, data extraction, statistical analysis and reporting will be performed in accordance with the Preferred Reporting Items for Systematic Reviews and Meta-Analyses (PRISMA) guidelines. Included papers will be full text articles, written between January 1980 and December 2019, comparing molecular characteristics of mpMRI-visible lesions and mpMRI-invisible lesions at the DNA, DNA-methylation, RNA, or protein level. Study bias and quality will be assessed using a modified Newcastle-Ottawa score. Additionally, we will conduct a novel bioinformatic analysis of supplementary material and publicly available data, to combine transcriptomic data and reveal common pathways highlighted across studies. To ensure methodological rigour, this protocol is written in accordance with the PRISMA Protocol 2015 checklist.

Ethics and dissemination

Ethical approval will not be required, as this is an academic review of published literature. Findings will be disseminated through publications in peer-reviewed journals, and presentations at national and international conferences.

PROSPERO registration number *CRD42019147423*

Strengths and limitations of this study:

- *This project will be the first comprehensive review of the molecular characteristics of mpMRI-visible and mpMRI-invisible lesions, following key the methodological steps of the PRISMA guidelines.*
- *Novel bioinformatic, over-representation analysis will reveal shared pathways and genes involved in prostate cancer mpMRI-conspicuity, that are overlooked by individual studies.*
- *The degree of heterogeneity between the included studies limits the comparability of derived results.*
- *There are relatively few studies that directly compare the genomic basis of mpMRI-visible and mpMRI-invisible lesions, which potentially limits the strength of conclusions from this work.*

For peer review only

BACKGROUND

Pre-biopsy multiparametric magnetic resonance imaging (mpMRI) has excellent diagnostic accuracy, and has improved risk stratification in prostate cancer diagnosis, compared to systematic transrectal ultrasound–guided prostate biopsy.[1,2] *Despite this, approximately 10-20% of clinically significant prostate cancers remain mpMRI invisible.*[1,3,4]

Suspicious mpMRI phenotypes appear to closely associate with the presence of prostate cancer, and to an extent, its Gleason grade.[1] *However, the degree to which the molecular landscape of prostate cancer correlates with mpMRI is unclear. In contrast, the general molecular basis of prostate cancer is well studied, with large-scale genomic enquires defining key drivers in tumour development and progression. Aberrations, including, recurrent mutations in SPOP1, FOXA1, IDH1, fusions in TMPRSS/ERG, ETV1/4, FLI1, and copy number alterations, such as MYC amplification or NKX3-1, RB1 and PTEN deletions or transcriptomic changes in AMACR, PCA3, GDF15 and MSMB, have all been investigated in the context of prostate cancer.*[5-7] *Over the past five years, there has been an increased effort to use this to develop our understanding of the genetic basis of prostate cancer conspicuity on mpMRI. It is now prudent to collate this growing evidence base, to clarify on progress made so far, and identify the most pertinent areas for future research.*

The aim of this systematic review and bioinformatic analysis is to determine and summarise for the first time, the genomic correlates of tumour visibility and invisibility on mpMRI, in order to elucidate the mechanisms that underpin mpMRI conspicuity, and the prognostic implication of mpMRI phenotypes.

For peer review only

METHODS AND ANALYSIS

This protocol of the planned systematic review and bioinformatic analysis is written in-line with the Preferred Reporting Items for Systematic Reviews and Meta-Analyses Protocol (PRISMA-P) 2015 checklist and is registered in the PROSPERO International Registry (PROSPERO Reg. No.: CRD42019147423).[8] Selected studies will be used for thematic synthesis and appropriate supplementary material extracted. In addition to the literature search, a bioinformatic approach will be used to identify relevant publicly available genomic data for a comprehensive analysis of over-represented pathways and biological functions.

Search methodology

Searches will be carried out using the MEDLINE, PubMed, EMBASE, and Cochrane databases in order to retrieve maximum yield of relevant evidence. The search will include Medical Subject Headings (MESH) terms and free text, combined with Boolean operators. The search will include the terms: 'prostate', 'cancer' and 'MRI', as well as multiple synonyms for the term 'genetics', to account for large heterogeneity in nomenclature and diversity of topics encompassed within this heading. Identified articles will be uploaded to Rayyan, a semi-automated tool to expedite the initial screening process and to allow two reviewers to filter duplicate studies and screen articles for relevance.[9] Furthermore, the reference section of all included articles will be searched manually to identify missed studies or additional data. Finally, experts will be consulted to identify additional literature. Only articles published between January 1980 and December 2019 will be included in our review. For the bioinformatic analysis, the National Center for Biotechnology Information (NCBI) Gene Expression Omnibus (GEO) and European Genome-phenome archive will be searched using the same search parameters as those used for the literature search. If sufficient processed data is available, data will be extracted from supplementary to ensure data veracity. In the case of missing data, the corresponding authors will be contacted directly.

Study selection and data extraction

Two researchers will independently screen eligible studies, assessing titles and abstracts for relevance. If considered eligible, the full text will be retrieved and further reviewed for eligibility. Any lack of concordance between reviewers will be discussed until a consensus is reached or passed to a third reviewer. Additionally, the reason for exclusion will be noted for later analysis. This process will be documented in detail in order to generate the PRISMA flow diagram.

Inclusion criteria

To be included in the analysis, studies must investigate one or more genomic aspects of the appearance of prostate cancer on mpMRI. Genomic investigation could be at the DNA level, investigating SNPs, or somatic alterations, or focused on larger scale alterations, such as copy number changes. Moreover, investigations revealing higher order structures, such as methylation, will also be included. Transcriptomic data analysing RNA expression (coding or non-coding) or microRNA will also be included, as will investigation into protein expression.

Exclusion criteria

Conference abstracts, correspondence articles, expert opinions, and case reports will be excluded. Studies that do not correlate mpMRI phenotypes with genomic data will be excluded. Articles focusing solely on macro-characteristics or clinical features of mpMRI conspicuity will be removed.

Data extraction

All extracted data will be collated in a dedicated data sheet. Both reviewers will extract data independently from each other and agree on consensus. Table 1 summarises data items to be collected.

Table 1 *Data collection items*

Item no.	***Data title***	***Data type***

For peer review only

1	*Year of publication*	*Study characteristic*
2	*Study authors*	*Study characteristic*
3	*Study design*	*Study characteristic*
4	*Patient population*	*Demographics*
5	*Number of participants*	*Demographics*
6	*mpMRI scoring scheme used*	*Methodology*
7	*Definition for clinically significant disease*	*Methodology*
8	*Definition for lesion visibility & invisibility*	*Methodology*
9	*Sample processing approach*	*Methodology*
10	*Biomolecule studied*	*Outcome*
11	*Differential expression of biomolecule*	*Outcome*

mpMRI, multiparametric magnetic resonance imaging; no., number.

Endpoints

The primary endpoint will be differential gene expression between mpMRI-visible and mpMRI-invisible lesions. Initial literature searching suggests that the majority of studies have focused on mRNA expression therefore, we will identify recurring genes which have a significant fold-change between visible and invisible lesions. Secondary endpoints will include explanatory links between gene function and mpMRI-conspicuity, and the prognostic value of differential gene enrichment. We will identify key themes within the literature, with a focus on: the MRI scoring systems used (e.g. PI-RADS, Likert, radiogenomic features), the criteria used to define lesion visibility (PI-RADS or Likert score thresholds) and the type of cohort used in the study (e.g. radical vs biopsy cohort). Additionally, we will identify which studies corrected for histopathological features such as lesion size and Gleason grade.

Sub-group analysis

A secondary analysis will look at whether gene panels, or individual genes, associated with mpMRI-conspicuity show different expression levels when key attributes (for example, Gleason grade and tumour size) are matched between study arms.

Statistical analysis

For the bioinformatic analysis, we will aim to keep data as close to the original format that was provided in the selected study. In the case of transcriptomic data, we will extract Log2 fold change and the associated false discovery rate (FDR) adjusted value between mpMRI-visible and mpMRI-invisible tumours. In more complex comparisons, we will attempt to extract details which indicate concordance of differential gene expression between studies, however, we will not include effect size if this is incomparable. If unavailable, we will simply compare highlighted genomic features and the direction of change between groups. Genes highlighted in multiple studies will be used (via over-representation analysis) to identify enriched pathways. This analysis will be performed using the WebGestalt: Gene Set Analysis Toolkit.[10] This method does not rely on effect size weighting and uses a modified Fisher's exact test to identify enriched biological processes.[11] Steps in the bioinformatic analysis are outlined in table 2.

Table 2 Steps in the bioinformatic analysis

Step no.	***Task***
Step 1	*Identifying studies with suitable supplementary data or associated data in data repositories*
Step 2	*Assessing comparability of results*
Step 3	*Comparing overlapping genes in multiple studies*
Step 4	*Over-representation analysis of genes present in multiple studies*
Step 5	*Comparison of suggested gene panels in independent cohort datasets*

No., number.

Risk of bias in individual studies

To assess bias and quality across the included studies, a modified Newcastle-Ottawa score will be used, designed to assess observational cohort studies.[12] *This scoring system is split into three main sections: selection, comparability, and outcome. Each section contains sub-questions that assess the quality of the research methodology, at the study level. Two reviewers will be involved with this process, and any disagreement will be settled by consensus. The results of the bias assessment will be used to influence data synthesis by providing an assessment of the reliability and applicability of the data produced. If studies are found to be of low quality (or high bias) then they may be excluded, or if included, they will be accompanied by appropriate commentary in the discussion. As this review centres around genomic studies of conspicuity as opposed to treatment outcome, non-applicable sections may be modified to reflect the evidence base and reduce reporting inaccuracy.*

For peer review only

DISCUSSION

In the UK, the National Institute for Health and Care Excellence (NICE) have recently updated their guidelines for men with suspected prostate cancer.[13] Men with suspected prostate cancer should now undergo mpMRI before prostate biopsy, as part of their risk stratification.[14] This move will likely see a rise in the numbers of mpMRI performed, and as such, we should strive to better understand the nature of disease detected and missed by this technology. Through systematic review and bioinformatic analysis, we aim to identify commonality between genomic-based studies which have investigated mpMRI-visible and mpMRI-invisible tumours. Our results will enhance the understanding of the genomic basis of prostate cancer visibility on mpMRI, the pathways that underpin this, and ultimately, the prognostic implication of disease conspicuity.

In our thematic analyses, we aim to derive the most prominent themes from studies that compare genetic differences in mpMRI-visible and mpMRI-invisible disease. The mechanistic links between genomic characteristics and the appearance of prostate cancer on mpMRI are not clear, however, early evidence suggests that genes controlling tumour cell proliferation may impact on perfusion, and as such, generation of mpMRI signal, particularly on diffusion weighted imaging (DWI) sequences.[15] Furthermore, combining suitable studies that link tumour conspicuity to genetic control will strengthen our understanding. Another important potential theme that we aim to elicit is the prognostic implication of different mpMRI-visibility or mpMRI-invisibility phenotypes (as defined by PI-RADS or Likert scoring schemes).[16] It appears that mpMRI-visible prostate cancer tends to have higher expression of genes associated with poor prognosis, however, this view is not unanimous throughout the literature and our rigorous systematic analysis will expose key differences in expression of prognostically significant genes, across different studies.[17,18]

In our bioinformatic analyses, we will compare large, publicly available genomic datasets that are linked to included studies from our systematic review. Described by Weidman and Arrison as 'data exploitation,' this approach allows effective use of collective information to obtain new insights.[19] We hope to elucidate commonalities between the strongest molecular differences between mpMRI-visible and mpMRI-invisible tumours. Pooling of data will strengthen association of particular genes with previously described features (for example, molecular pathways and prognostic value). Furthermore, bioinformatic comparison of these datasets may reveal potential key targets for future research, including the development of peripheral biomarkers to identify clinically significant, mpMRI-inconspicuous prostate cancer. A potential limitation may be data heterogeneity, due to differences in definitions for MRI-visibility and methods of identifying genetic determinants. Our analysis will attempt to overcome this issue by focusing on studies with similar definitions of visibility, and restricting our analyses to identified genes and enriched pathways, as opposed to effect sizes.

In summary, this systematic review and novel bioinformatic analysis of publicly available genomic data will collate extant evidence in this emerging field, for the first time. Synthesis of these studies will enhance our current understanding of the role that genetics play in the mpMRI-conspicuity of prostate cancer, and will clarify the most pertinent areas for future research.

For peer review only

Trial status

- *Preliminary searches: started.*
- *Piloting of the study selection process: started.*
- *Formal screening: started.*
- *Data extraction: not started.*
- *Risk of bias assessment: not started.*
- *Data analysis: not started.*

Draft of search strategy for MEDLINE, EMBASE, PubMed and Cochrane Databases

(prostate AND cancer) AND (gene OR genetic OR genome OR genomic OR transcriptome OR transcriptomic OR epigenetic) AND (magnetic resonance imaging OR MRI)

Acknowledgements

None.

Patient and public involvement

Patients and the public were not involved in the development of this systematic review protocol.

For peer review only

ETHICS AND DISSEMINATION

Due to the nature of the study, there are no relevant ethical concerns and informed consent will not be required. The protocol and systematic review will be disseminated via a peer-reviewed journal.

Author affiliations

[1] UCL Division of Surgery and Interventional Science, University College London, London, UK

[2] London Deanery of Urology, London, UK

[3] Department of Urology, University College London Hospitals NHS Foundation Trust, London, UK

[4] UCL Cancer Institute, University College London, London, UK

[5] Department of Urology, Frimley Health NHS Foundation Trust, London, UK

[6] Department of Radiology, University College London Hospitals NHS Foundation Trust, London, UK

[7] Department of Pathology, University College London Hospitals NHS Foundation Trust, London, UK

[8] School of Healthcare Sciences, Cardiff University, Wales, UK

Contributors

The authors' contribution includes, but is not limited to, the following: JMN and BSS drafted the manuscript and created the study concept. MAP gave advice on genetic aspects. CA, RB, AK, AF, DK, VK, HW and ME provided supervision and guidance during the study. All authors reviewed and approved the manuscript in its current form.

Funding

Joseph M. Norris is funded by the Medical Research Council (MRC) on an MRC Clinical Research Training Fellowship (MRC Grant Ref: MR/S00680X/1). Benjamin S. Simpson receives funding from the Rosetrees Foundation.

Competing interests

None declared.

Patient consent for publication

Not required.

Provenance and peer review

Not commissioned; externally peer-reviewed.

Open access

This is an Open Access article distributed in accordance with the Creative Commons Attribution Non Commercial (CC BY-NC 4.0) license, which permits others to distribute, remix, adapt, build upon this work non-commercially, and license their derivative works on different terms, provided the original work is properly cited and the use is non-commercial. See: http://creativecommons.org/ licenses/by-nc/4.0/

© Article author(s) (or their employer(s) unless otherwise stated in the text of the article) 2019. All rights reserved. No commercial use is permitted unless otherwise expressly granted.

For peer review only

REFERENCES

1. Ahmed HU, El-Shater Bosaily A, Brown LC, Gabe R, Kaplan R, Parmar MK, Collaco-Moraes Y, Ward K, Hindley RG, Freeman A, Kirkham AP, Oldroyd R, Parker C, Emberton M; PROMIS study group. Diagnostic accuracy of multi-parametric MRI and TRUS biopsy in prostate cancer (PROMIS): a paired validating confirmatory study. Lancet. 2017 Feb 25;389(10071):815-22.
2. Kasivisvanathan V, Rannikko AS, Borghi M, Panebianco V, Mynderse LA, Vaarala MH, Briganti A, Budäus L, Hellawell G, Hindley RG, Roobol MJ, Eggener S, Ghei M, Villers A, Bladou F, Villeirs GM, Virdi J, Boxler S, Robert G, Singh PB, Venderink W, Hadaschik BA, Ruffion A, Hu JC, Margolis D, Crouzet S, Klotz L, Taneja SS, Pinto P, Gill I, Allen C, Giganti F, Freeman A, Morris S, Punwani S, Williams NR, Brew-Graves C, Deeks J, Takwoingi Y, Emberton M, Moore CM; PRECISION Study Group Collaborators. MRI-targeted or standard biopsy for prostate-cancer diagnosis. N Engl J Med. 2018 May 10;378(19):1767-77.
3. Radtke JP, Kuru TH, Boxler S, Alt CD, Popeneciu IV, Huettenbrink C, Klein T, Steinemann S, Bergstraesser C, Roethke M, Roth W, Schlemmer HP, Hohenfellner M, Hadaschik BA. Comparative analysis of transperineal template saturation prostate biopsy versus magnetic resonance imaging targeted biopsy with magnetic resonance imaging-ultrasound fusion guidance. J Urol. 2015 Jan;193(1):87-94.
4. Drost FH, Osses DF, Nieboer D, Steyerberg EW, Bangma CH, Roobol MJ, Schoots IG. Prostate MRI, with or without MRI-targeted biopsy, and systematic biopsy for detecting prostate cancer. Cochrane Database Syst Rev. 2019 Apr 25;4:CD012663.
5. Taylor BS, Schultz N, Hieronymus H, Gopalan A, Xiao Y, Carver BS, Arora VK, Kaushik P, Cerami E, Reva B, Antipin Y. Integrative genomic profiling of human prostate cancer. Cancer Cell. 2010 Jul 13;18(1):11-22.
6. Ross-Adams H, Lamb AD, Dunning MJ, Halim S, Lindberg J, Massie CM, Egevad LA, Russell R, Ramos-Montoya A, Vowler SL, Sharma NL, Kay J, Whitaker H, Clark J, Hurst R, Gnanapragasam VJ, Shah NC, Warren AY, Cooper CS, Lynch AG, Stark R, Mills IG, Grönberg H, Neal DE; CamCaP Study Group. Integration of copy number and transcriptomics provides risk stratification in prostate cancer: a discovery and validation cohort study. EBioMedicine. 2015 Jul 29;2(9):1133-44.
7. Abeshouse A, Ahn J, Akbani R, Ally A, Amin S, Andry CD, Annala M, Aprikian A, Armenia J, Arora A, Auman JT. The molecular taxonomy of primary prostate cancer. Cell. 2015 Nov 5;163(4):1011-25.
8. Moher D, Shamseer L, Clarke M, Ghersi D, Liberati A, Petticrew M, Shekelle P, Stewart LA; PRISMA-P Group. Preferred reporting items for systematic review and meta-analysis protocols (PRISMA-P) 2015 statement. Syst Rev. 2015 Jan 1;4:1.
9. Ouzzani M, Hammady H, Fedorowicz Z, Elmagarmid A. Rayyan—a web and mobile app for systematic reviews. 2016 Dec 5;5(1):210.
10. Liao Y, Wang J, Jaehnig EJ, Shi Z, Zhang B. WebGestalt 2019: gene set analysis toolkit with revamped UIs and APIs. Nucleic Acids Res. 2019 Jul 2;47(W1):W199-205.
11. Khatri P, Sirota M, Butte AJ. Ten years of pathway analysis: current approaches and outstanding challenges. PLoS Comput Biol. 2012;8:e1002375.
12. Wells G, Shea B, Oconnell D . The Newcastle-Ottawa scale (NOS) for assessing the quality of nonrandomized studies in meta-analyses. Available from: http://www.ohri.ca/programs/clinical_epidemiology/oxford.asp
13. Wise J. NICE recommends MRI for suspected prostate cancer to reduce biopsies. BMJ. 2018; 363:k5290.
14. Dasgupta P, Davis J, Hughes S. NICE guidelines on prostate cancer 2019. BJU Int. 2019 Jul;124(1):1.
15. Li P, You S, Nguyen C, Wang Y, Kim J, Sirohi D, Ziembiec A, Luthringer D, Lin SC, Daskivich T, Wu J, Freeman MR, Saouaf R, Li D, Kim HL. Genes involved in prostate cancer progression determine MRI visibility. Theranostics. 2018 Feb 12;8(7):1752-65.
16. Padhani AR, Barentsz J, Villeirs G, Rosenkrantz AB, Margolis DJ, Turkbey B, Thoeny HC, Cornud F, Haider MA, Macura KJ, Tempany CM, Verma S, Weinreb JC. PI-RADS steering committee: the PI-RADS multiparametric MRI and MRI-directed biopsy pathway. Radiology. 2019 Aug;292(2):464-7
17. Houlahan KE, Salmasi A, Sadun TY, Pooli A, Felker ER, Livingstone J, Huang V, Raman SS, Ahuja P, Sisk AE Jr, Boutros PC, Reiter RE. Molecular hallmarks of multiparametric magnetic resonance imaging visibility in prostate cancer. Eur Urol. 2019 Jul;76(1):18-23.
18. Parry MA, Srivastava S, Ali A, Cannistraci A, Antonello J, Barros-Silva JD, Ubertini V, Ramani V, Lau M, Shanks J, Nonaka D, Oliveira P, Hambrock T, Leong HS, Dhomen N, Miller C, Brady G, Dive C, Clarke NW, Marais R, Baena E. Genomic evaluation of multiparametric magnetic resonance imaging-visible and -nonvisible lesions in clinically localised prostate cancer. Eur Urol Oncol. 2019 Feb;2(1):1-11.
19. Weidman S, Arrison T. Steps toward large-scale data integration in the sciences: summary of a workshop. National Research Council of the National Academies. 2010.

For peer review only

For peer review only

PRISMA-P (Preferred Reporting Items for Systematic review and Meta-Analysis Protocols) 2015 checklist: recommended items to address in a systematic review protocol*

	Section and topic	Item No	Checklist item
	**ADMINISTRATIVE INFORMATION**		
	Title:		
Page 1 ✓	Identification	1a	Identify the report as a protocol of a systematic review
	Update	1b	If the protocol is for an update of a previous systematic review, identify as such
	Registration	2	If registered, provide the name of the registry (such as PROSPERO) and registration number
	Authors:		
Page 1 ✓	Contact	3a	Provide name, institutional affiliation, e-mail address of all protocol authors; provide physical mailing address of corresponding author
	Contributions	3b	Describe contributions of protocol authors and identify the guarantor of the review
n/a ✓	Amendments	4	If the protocol represents an amendment of a previously completed or published protocol, identify as such and list changes; otherwise, state plan for documenting important protocol amendments
	Support:		
Page 1 ✓	Sources	5a	Indicate sources of financial or other support for the review
	Sponsor	5b	Provide name for the review funder and/or sponsor
	Role of sponsor or funder	5c	Describe roles of funder(s), sponsor(s), and/or institution(s), if any, in developing the protocol
	**INTRODUCTION**		
Page 3 ✓	Rationale	6	Describe the rationale for the review in the context of what is already known
	Objectives	7	Provide an explicit statement of the question(s) the review will address with reference to participants, interventions, comparators, and outcomes (PICO)
	**METHODS**		
Page 4 ✓	Eligibility criteria	8	Specify the study characteristics (such as PICO, study design, setting, time frame) and report characteristics (such as years considered, language, publication status) to be used as criteria for eligibility for the review
Page 4 ✓	Information sources	9	Describe all intended information sources (such as electronic databases, contact with study authors, trial registers or other grey literature sources) with planned dates of coverage
Page 9 ✓	Search strategy	10	Present draft of search strategy to be used for at least one electronic database, including planned limits, such that it could be repeated
Page 4 ✓	Study records:		
	Data management	11a	Describe the mechanism(s) that will be used to manage records and data throughout the review

For peer review only

Pages 4 & 5 ✓	Selection process	11b	State the process that will be used for selecting studies (such as two independent reviewers) through each phase of the review (that is, screening, eligibility and inclusion in meta-analysis)
	Data collection process	11c	Describe planned method of extracting data from reports (such as piloting forms, done independently, in duplicate), any processes for obtaining and confirming data from investigators
	Data items	12	List and define all variables for which data will be sought (such as PICO items, funding sources), any pre-planned data assumptions and simplifications
Pages 5 & 6 ✓	Outcomes and prioritization	13	List and define all outcomes for which data will be sought, including prioritization of main and additional outcomes, with rationale
Pages 6 & 7 ✓	Risk of bias in individual studies	14	Describe anticipated methods for assessing risk of bias of individual studies, including whether this will be done at the outcome or study level, or both; state how this information will be used in data synthesis
Page 6 ✓	Data synthesis	15a	Describe criteria under which study data will be quantitatively synthesised
		15b	If data are appropriate for quantitative synthesis, describe planned summary measures, methods of handling data and methods of combining data from studies, including any planned exploration of consistency (such as I^2, Kendall's τ)
		15c	Describe any proposed additional analyses (such as sensitivity or subgroup analyses, meta-regression)
		15d	If quantitative synthesis is not appropriate, describe the type of summary planned
Pages 6 & 7 ✓	Meta-bias(es)	16	Specify any planned assessment of meta-bias(es) (such as publication bias across studies, selective reporting within studies)
	Confidence in cumulative evidence	17	Describe how the strength of the body of evidence will be assessed (such as GRADE)

*** It is strongly recommended that this checklist be read in conjunction with the PRISMA-P Explanation and Elaboration (cite when available) for important clarification on the items. Amendments to a review protocol should be tracked and dated. The copyright for PRISMA-P (including checklist) is held by the PRISMA-P Group and is distributed under a Creative Commons Attribution Licence 4.0.**

From: Shamseer L, Moher D, Clarke M, Ghersi D, Liberati A, Petticrew M, Shekelle P, Stewart L, PRISMA-P Group. Preferred reporting items for systematic review and meta-analysis protocols (PRISMA-P) 2015: elaboration and explanation. BMJ. 2015 Jan 2;349(jan02 1):g7647.

For peer review only
